# Involvement of JNK/NFκB Signaling Pathways in the Lipopolysaccharide-Induced Modulation of Aquaglyceroporin Expression in 3T3-L1 Cells Differentiated into Adipocytes

**DOI:** 10.3390/ijms17101742

**Published:** 2016-10-18

**Authors:** Jeanne Durendale Chiadak, Tatjana Arsenijevic, Francoise Gregoire, Nargis Bolaky, Valerie Delforge, Jason Perret, Christine Delporte

**Affiliations:** 1 Laboratory of Pathophysiological and Nutritional Biochemistry, Université Libre de Bruxelles, 1070 Brussels, Belgium; jeanne.chiadak@ulb.ac.be (J.D.C.); tarsenijevic@yahoo.com (T.A.); francoise.gregoire@ulb.ac.be (F.G.); nargis.bolaky@ulb.ac.be (N.B.); valerie.delforge@yahoo.fr (V.D.); Jason.Perret@ulb.ac.be (J.P.); 2 Department of Biochemistry, Faculty of Sciences, University of Dschang, 67 Dschang, Cameroon

**Keywords:** obesity, glycerol, aquaglyceroporins, adipocytes, pathways

## Abstract

Aquaglyceroporins, belonging to the family of aquaporins (AQPs), are integral plasma membrane proteins permeable to water and glycerol that have emerged as key players in obesity. The aim of this study was to investigate the expression profile of AQPs in undifferentiated and differentiated 3T3-L1 cells and to investigate the changes in expression of aquaglyceroporins in 3T3-L1 cells differentiated into adipocytes and subjected to lipopolysaccharide (LPS) mimicking inflammation occurring during obesity. Furthermore, the study aimed at identifying the signaling cascade involved in the regulation of aquaglyceroporins expression upon LPS stimulation. 3T3-L1 cells were grown as undifferentiated cells (UDC; preadipocytes) or cells differentiated into adipocytes (DC, adipocytes). DC were incubated in the presence or absence of LPS with or without inhibitors of various protein kinases. AQPs mRNA expression levels were measured by real-time quantitative polymerase chain reaction (RT-qPCR). AQP1, AQP2, AQP3, AQP9 and AQP11 mRNA were expressed in both UDC and DC, whereas AQP4, AQP7 and AQP8 mRNA were expressed only in DC. In DC, LPS up-regulated AQP3 mRNA levels (*p* < 0.05) compared to control; these effects were inhibited by CLI095, SP600125 and BAY11-7082 (*p* < 0.05). LPS decreased both AQP7 and AQP11 mRNA levels (*p* < 0.01) in DC as compared to control; this decrease was inhibited by CLI095 and BAY11-7082 (*p* < 0.05) and additionally by SP00125 for AQP7 (*p* < 0.05). SB203580 had no effect on LPS-induced AQP3, AQP7 and AQP11 mRNA levels modulations. In conclusion, our results clearly show that many AQPs are expressed in murine 3T3-L1 adipocytes. Moreover, in DCs, LPS led to decreased AQP7 and AQP11 mRNA levels but to increased AQP3 mRNA levels, resulting from the Toll-like receptor 4 (TLR4)-induced activation of JNK and/or NFκB pathway.

## 1. Introduction

The incidence of obesity worldwide is increasing constantly and represents a critical risk factor for the onset of various diseases such as cardiovascular diseases, diabetes mellitus, hyperlipidemia and cardiac infarction [1,2]. The increase of adipose tissue mass that accompanies obesity is due to an expansion in adipocyte number (hyperplasia) and size (hypertrophy) [3]. Adipocytes play a crucial role in whole-body energy homeostasis as it supplies energy during starvation. However, over-nutrition and lack of exercise result in over-accumulation of fat [4]. Glycerol plays a major role in energy homeostasis, as it represents the carbon backbone of triacylglycerol (TAG) and constitutes the major substrate for hepatic gluconeogenesis during fasting [5,6]. Circulating plasma glycerol results from its release from TAG during lipolysis and its reabsorption by kidney proximal tubules [7,8].

Aquaporins (AQPs) are a family of transmembrane water channels involved in transcellular water transport [9,10]. To date, 13 mammalian AQPs have been identified: AQP0 to AQP12 [9,10]. AQPs can be subdivided into: (i) classical AQPs, only permeable to water (AQP1, AQP2, AQP4, AQP5, AQP6, AQP8) [9,10]; (ii) aquaglyceroporins, permeable to glycerol in addition to water (AQP3, AQP7, AQP9, AQP10) [11,12]; (iii) non-classical AQPs, presenting slightly different structural features in the conserved motifs and of debated permeability (AQP11, AQP12) [13]. Recently, AQP11, though still considered as a non-classical AQP, has been shown to be permeable to glycerol and therefore may be considered as an additional aquaglyceroporin [14]. Besides their water transport function, AQPs have also been involved in a series of other cell functions, such as in cell migration and proliferation [15,16,17,18,19,20]. Aquaglyceroporins have emerged as key players in obesity and development of insulin resistance [21,22]. The main function of aquaglyceroporins in adipocytes is the control of glycerol uptake and release, two key steps for TAG synthesis (lipogenesis) and hydrolysis (lipolysis), respectively [21]. Indeed, adipose tissue constitutes the most important source of plasma glycerol [7]. While AQP7 was considered as the sole aquaglyceroporin in adipose tissue for many years, it has been recently shown that AQP3, AQP9 and AQP11 are also expressed and represent novel additional pathways for the transport of glycerol in human adipocytes [14,23,24]. In addition, AQP10 represents a pathway for glycerol efflux in human adipocytes [25]. It must, however, be noted that *Aqp10* gene is a pseudogene in mice [26]. 

Links between aquaglyceroporins expression and obesity have been established [23,27,28,29,30]. Studies conducted in *Aqp7* null mice have linked the absence of AQP7 expression to the development of obesity and adipocyte hypertrophy [31,32]. *Aqp7* deficiency leads to glycerol retention within adipose tissue, ultimately leading to acceleration of TAG synthesis and accumulation in mice adipocytes [31,32]. Obesity is associated with increased AQP3 and AQP9 expression and decreased AQP7 expression in human subcutaneous adipose tissue [24]. Aquaglyceroporins display different subcellular localization in murine 3T3-L1 adipocytes. Indeed, AQP3 is present in the plasma membrane and cytoplasm and AQP7 resides predominantly in the cytoplasm, while both translocate to the plasma membrane upon hormone-induced increase in cAMP [24]. In addition, AQP9 is constitutively expressed in the plasma membrane and does not appear to undergo translocation upon hormone stimulation [24]. Finally, AQP11 is primarily located intracellularly in the vicinity of lipid droplets [14]. Despite previous studies indicating AQP11 trafficking to the plasma membrane in oocytes [33] and Chinese hamster ovary (CHO) cells [34], additional studies are necessary to investigate possible hormone-induced regulatory mechanism that might induce AQP11 translocation to the plasma membrane in adipocytes.

Low level elevations of gut derived endotoxin (lipopolysaccharide (LPS)) have been shown to play an important role in obesity [35], similarly to the consequential production of proinflammatory cytokines (such as interleukin-6 and tumor necrosis α (TNFα)) [36], resulting from LPS-induced TLR4 activation. Previous studies have shown that LPS, mimicking inflammation occurring during obesity, can affect the expression of several aquaglyceroporins. Indeed, LPS down-regulated AQP3 levels in human colon epithelial cells through a p38/JNK signaling pathway [37], but enhanced AQP9 expression in rat brain by an, as yet, uncharacterized signaling pathway [38]. However, it is currently unknown if LPS affects aquaglyceroporin expression in adipocytes. The aim of this study was to determine the AQPs expression profile in undifferentiated 3T3-L1 cells (UDC; preadipocytes) vs. 3T3-L1 cells differentiated into adipocytes (DC; adipocytes), as well as the modulation of aquaglyceroporins expression by LPS in the DC. In addition, we aimed at identifying the signaling cascades involved in such modulation.

## 2. Results

### 2.1. Expression of Aquaporins (AQPs) mRNA in 3T3-L1 Cells Differentiated into Adipocytes (DC; Adipocytes) vs. Undifferentiated 3T3-L1 Cells (UDC; Preadipocytes)

To investigate possible differential expression of AQPs between the undifferentiated 3T3-L1 cells (UDC; preadipocytes) and 3T3-L1 cells differentiated into adipocytes (DC; adipocytes), the AQPs mRNA levels were measured by real-time quantitative polymerase chain reaction (RT-qPCR) both in the UDC and the DC. As the *Aqp10* gene is a pseudogene [26], AQP10 was not included in the study of the expression of AQPs in UDC and DC. UDC expressed AQP1, AQP2, AQP3, AQP9 and AQP11 mRNA as shown by their respective cycle threshold (*C*_t_) of 29.24 ± 0.81, 35.96 ± 1.86, 33.54 ± 1.11, 36.25 ± 0.72, 30.92 ± 0.13, respectively (*n* = 3; Table 1). AQP4, AQP5, AQP6, AQP7, AQP8 and AQP12 were undetectable in UDC (Table 1). In DC, AQP1, AQP2, AQP3, AQP4, AQP7, AQP8, AQP9 and AQP11 mRNA were expressed and their *C*_t_ were 24.86 ± 0.03, 35.40 ± 0.96, 31.34 ± 0.06, 31.04 ± 0.97, 24.66 ± 0.09, 27.66 ± 0.45, 36.24 ± 0.67, 28.82 ± 0.12, respectively (*n* = 3, Table 1). AQP2, AQP3 and AQP9 mRNA levels remained unchanged upon differentiation. In contrast, AQP1, AQP7, AQP8 and AQP11 mRNA were significantly increased in DC as compared to UDC (*p* < 0.05). AQP5, AQP6 and AQP12 could not be detected in DC.

### 2.2. Effect of Lipopolysaccharide (LPS) on AQP3, AQP7 and AQP11 mRNA Levels in DC

**Figure 1:**
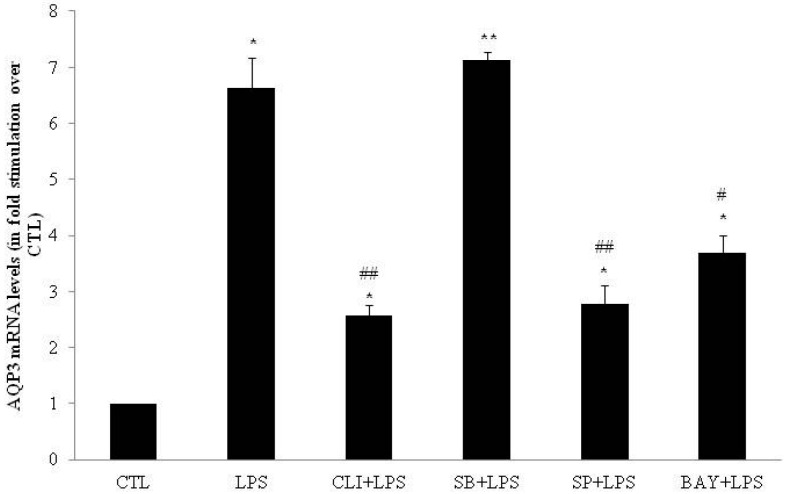
mRNA expression of aquaporin-3 (AQP3) in 3T3-L1 cells differentiated into adipocytes (DC; adipocytes) treated with lipopolysaccharide (LPS) in the presence or absence of inhibitors. DC were treated for 4 h with water (CTL) or 4 h with 1 µg/mL LPS (LPS). DC were also pretreated for 1 h with 20 µmol/L SB203580 (p38 MAPK inhibitor), 50 µmol/L, SP600125 (JNK inhibitor), 10 µmol/L BAY11-7082 (IκB-α inhibitor), or 3 µmol/L CLI-095 (TLR4 signaling inhibitor) prior to 4 h CTL or LPS treatment in the presence of inhibitor. Relative mRNA levels were determined as described under Material and Methods. The results are expressed as mRNA levels (in fold stimulation over CTL set to 1) and are the means ± S.E.M. of 3 independent experiments. Data were analyzed using *t*-test for unique sample and repeated measure ANOVA, * *p* < 0.05, ** *p* < 0.01 vs. CTL; # *p* < 0.05, ## *p* < 0.01 vs. LPS.

**Figure 2:**
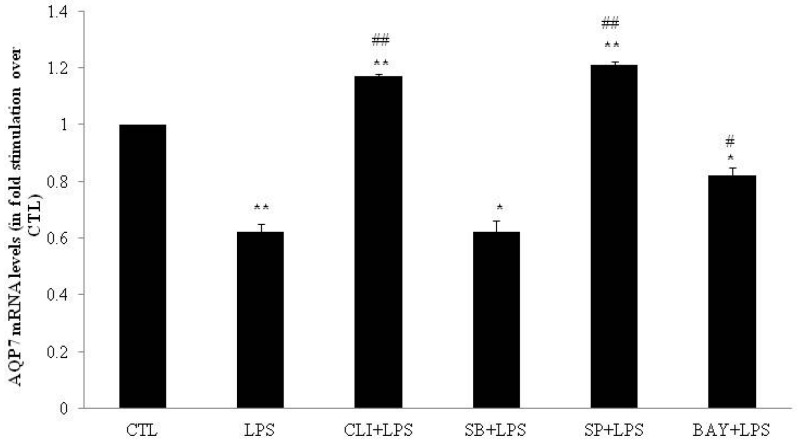
mRNA expression of aquaporin-7 (AQP7) in DC treated with LPS in the presence or absence of inhibitors. DC were treated for 4 h with water (CTL) or 4 h with 1 µg/mL LPS (LPS). DC were also pretreated for 1 h with 20 µmol/L SB203580 (p38 MAPK inhibitor), 50 µmol/L, SP600125 (JNK inhibitor), 10 µmol/L BAY11-7082 (IκB-α inhibitor), or 3 µmol/L CLI-095 (TLR4 signaling inhibitor) prior to 4 h CTL or LPS treatment in the presence of inhibitor. Relative mRNA levels were determined as described under Material and Methods. The results are expressed as mRNA levels (in fold stimulation over CTL set to 1) and are the means ± S.E.M. of three independent experiments. Data were analyzed using *t-*test for unique sample and repeated measure ANOVA, * *p* < 0.05, ** *p* < 0.01 vs. CTL; # *p* < 0.05, ## *p* < 0.01 vs. LPS.

**Figure 3:**
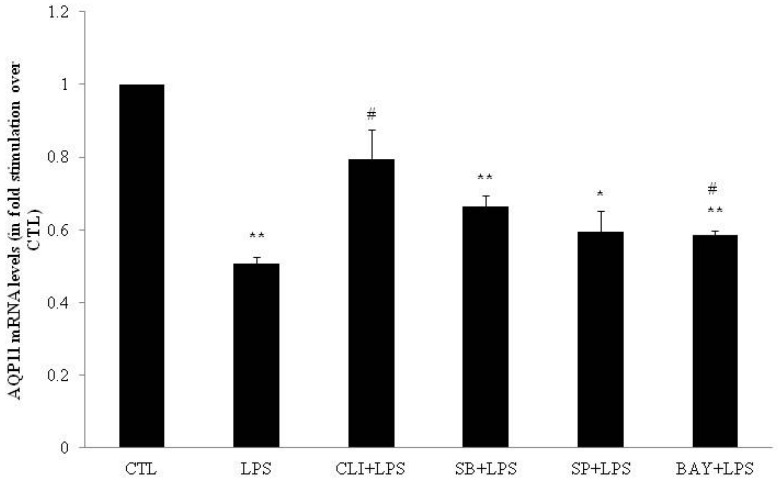
mRNA expression of aquaporin-11 (AQP11) in differentiated 3T3-L1 cells treated with LPS in the presence or absence of inhibitors. DC were treated for 4h with water (CTL) or 4 h with 1 µg/mL LPS (LPS). DC were also pretreated for 1 h with 20 µmol/L SB203580 (p38 MAPK inhibitor), 50 µmol/L, SP600125 (JNK inhibitor), 10 µmol/L BAY11-7082 (IκB-α inhibitor), or 3 µmol/L CLI-095 (TLR4 signaling inhibitor) prior to 4 h CTL or LPS treatment in the presence of inhibitor. Relative mRNA levels were determined as described under Material and Methods. The results are expressed as mRNA levels (in fold stimulation over CTL set to 1) and are the means ± S.E.M. of three independent experiments. Data were analyzed using *t*-test for unique sample and repeated measure ANOVA, * *p* < 0.05, ** *p* < 0.01 vs. CTL; **#** *p* < 0.05 vs. LPS.

In DC, AQP3 mRNA levels were increased seven-fold by LPS as compared to water (control condition; CTL) (*p* < 0.05; Figure 1). In contrast, LPS decreased both AQP7 (0.62-fold; *p* < 0.01; Figure 2) and AQP11 (0.50-fold; *p* < 0.01; Figure 3) mRNA levels as compared to CTL.

### 2.3. Effect of TLR4 Inhibition on AQP3, AQP7 and AQP11 mRNA Levels in DC

To determine whether the LPS-induced modulations of AQP3, AQP7 and AQP11 mRNA levels were mediated via TLR4 receptors, DC were pretreated for one hour with 3 µmol/L of the TLR4 signaling inhibitor CLI-095 and then co-incubated with LPS (1 µg/mL) for four hours. CLI-095 significantly decreased by 64% the LPS-induced AQP3 mRNA levels increase (*p* < 0.01; Figure 1). CLI-095 significantly reversed LPS-induced decrease in AQP7 and AQP11 mRNA levels by 188% (*p* < 0.01) and 157% (*p* < 0.05), respectively (Figure 2 and Figure 3).

### 2.4. Involvement of p38, JNK and NFκB Signaling Pathways on AQP3, AQP7 and AQP11 mRNA Levels in DC

To determine whether the LPS-induced modulations of AQP3, AQP7 and AQP11 mRNA levels involved p38, JNK or NFκB pathway, DC were treated for one hour with 20 µmol/L of p38 inhibitor SB203580, 50 µmol/L of JNK inhibitor SP600125 or 10 µmol/L of IκB-α inhibitor BAY11-7082 prior to co-incubation for four hours with LPS. SB203580 had no effect on LPS-induced modulation of AQP3, AQP7 and AQP11 mRNA levels (Figure 1, Figure 2 and Figure 3). SP600125 and BAY11-7082 inhibited LPS-induced increase in AQP3 mRNA levels by 61% (*p* < 0.01) and 48% (*p* < 0.05), respectively (Figure 1). SP600125 and BAY11-7082 reversed LPS-induced decrease in AQP7 mRNA levels by 195% (*p* < 0.01) and 132% (*p* < 0.05), respectively (Figure 2). Finally, BAY11-7082 reversed LPS-induced decrease in AQP11 mRNA levels by 115% (*p* < 0.05) (Figure 3).

## 3. Discussion

Aquaglyceroporins ensure the control of glycerol uptake and release in adipocytes—two key steps for TAG synthesis (lipogenesis) and hydrolysis (lipolysis), respectively [21]. Human adipocytes have been shown to express AQP7 [32,39,40], as well as AQP3, AQP9 and AQP11 [14,23,24]. The aims of our study were to determine the AQPs expression profile in murine adipocytes, and to understand the regulation of aquaglyceroporins expression under LPS treatment mimicking inflammation typically occurring during obesity.

This study reports for the first time the complete AQPs expression profile in UDC and DC. Our data show detectable expression of AQP1, AQP2, AQP3, AQP9 and AQP11 mRNA in UDC and DC. The expression of AQP1 and AQP11 were significantly higher in DC (adipocytes) than in UDC (predipocytes), i.e., post adipocyte differentiation induction. In contrast, the expression of AQP2, AQP3 and AQP9 remained comparable in DC with respect to UDC. In addition, the appearance of AQP4, AQP7 and AQP8 expression was observed in DC. Our data are in agreement with previous works reporting the expression of AQP3, AQP7 and AQP9 in DC [24], as well as the undetectable expression of AQP7 in UDC and detectable expression of AQP7 in DC [41,42]. However, our data diverge from previously published data concerning AQP11 expression, which has been only detected in DC and not in UDC [14], and AQP5 expression, which has been detected in both UDC and DC [43]. The apparent discrepancies between our data and previously published studies might results from different culture conditions as well as methodologies used for the detection of AQP expression. Nevertheless, where AQP11 is concerned, both our data and previous studies [14] showed an increase in its expression in DC as compared to UDC. AQP11, classified as a non-classical AQP with debated permeability [13], has recently been shown to be permeable to glycerol [14]. Additional studies will be necessary to investigate hormone-induced regulatory mechanism that could induce AQP11 trafficking in adipocytes. The presence of both AQP1 and AQP2 in UDC, and of AQP1, AQP2 and AQP8 in DC has not been previously reported but their roles remain to be addressed by further investigations. In addition, the reason for multiple aquaglyceroporins expression in adipocyte remains poorly understood despite their differential regulation [24,44]. Nevertheless, regulatory mechanisms controlling aquaglyceroporins subcellular localization and trafficking may play an important role in the subtle control of glycerol metabolism involved in the control of energy homeostasis in adipocytes [5,6]. Finally, further studies need to be carried out to investigate the time course of AQPs expression during the differentiation process in order to better understand the physiological roles AQPs may eventually play in this process.

**Figure 4:**
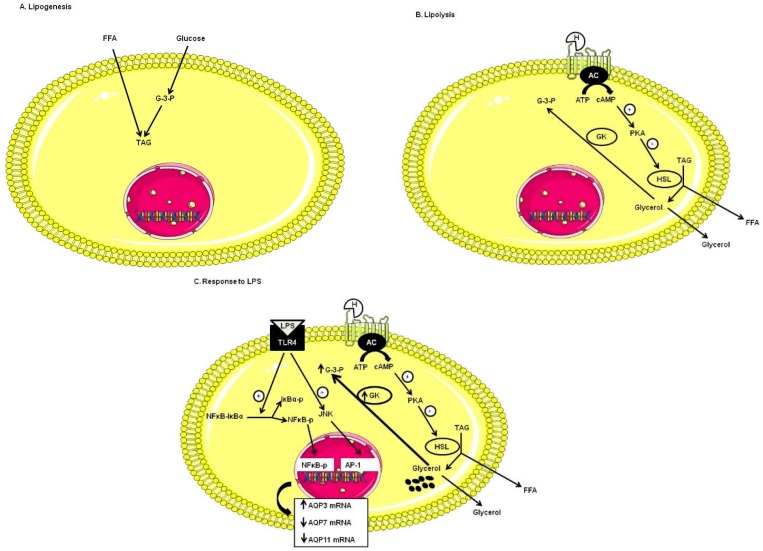
Data summary showing how LPS induced AQP3, AQP7 and AQP11 modulations in DC. (**A**) Lipogenesis: upon feeding, glucose and free fatty acid produce triacylglycerol that are stored in adipocytes; (**B**) Lipolysis: when the energy balance becomes negative, such as upon fasting and exercise, triacylglycerol is hydrolyzed to glycerol and free fatty acids; (**C**) Response to LPS: in the obese state induced by LPS in DC, AQP3 and AQP7 mRNA are modulated via TLR-4 signaling with activation of the JNK and NFκB pathways, whereas AQP11 mRNA is down-regulated via TLR-4 signaling with activation of NFκB pathway only. A mechanism for progressive triacylglycerol accumulation in adipocytes of *Aqp7*-deficient mice has been proposed: reduced plasma membrane glycerol permeability would result in an increased intracellular glycerol concentration that would activate glycerol kinase, increase glycerol-3-phosphate and hence triacylglycerol biosynthesis [31,32]. Abbreviations: TAG: triacylglycerol, G-3-P: glycerol-3-phosphate, FFA: free fatty acids, H: catecholamines or hormones, AC: adenyl cyclase, ATP: adenosine triphosphate, cAMP: cyclic adenosine monophosphate, PKA: protein kinase A, HSL: hormono sensitive lipase, GK: glycerol kinase, LPS: lipopolysaccharide, TLR-4: Toll-like receptor 4, IκBα: inhibitor of kappa B, NFκB: nuclear factor-κB, JNK: c-Jun N-terminal kinases, AP-1: Activated protein 1, AQP: Aquaporins.

In this study, we also aimed at studying the modulation of aquaglyceroporins expression upon LPS stimulation mimicking inflammation encountered in obese state, and identifying signaling pathways involved in such processes. As AQP9 mRNA levels were very low and also unmodified upon LPS stimulation in DC, we focused on the regulation of AQP3, AQP7 and AQP11 expression. In response to LPS, there was an increase in AQP3 mRNA levels, and a decrease in both AQP7 and AQP11 mRNA levels. These LPS-induced aquaglyceroporins modulations were inhibited by a TLR4 inhibitor, thereby suggesting the involvement of the TLR4-signaling pathway in these processes. LPS-induced increase in AQP3 expression corroborated increased AQP3 expression detected in subcutaneous adipose tissue from obese subjects [24]. Similarly, decreased AQP7 expression was observed in response to LPS in our study, corroborating similar results observed in human adipose tissue from obese subjects [23,27,28,29,30]. Progressive TAG accumulation in adipocytes of *Aqp7*-deficient mice resulted from reduced plasma membrane glycerol permeability and subsequent increase in intracellular glycerol concentration, which would activate glycerol kinase and increase glycerol-3-phosphate concentration and hence TAG biosynthesis [31,32] (Figure 4). Upon fasting, glycerol secretion was not completely abolished in *Aqp7*-depleted adipocytes [31,45], suggesting that other transporters must be involved in glycerol transport. In addition, *Aqp7* deficiency was shown to be associated with insulin-resistance in mice [31]. In contrast, overexpression of AQP7 improved insulin resistance in dexamethasone and TNFα-treated adipocytes [46] and polyphenol-induced increase in AQP7 expression led to the inhibition of adipocyte hypertrophy in diet-induced obesity in rats [47]. AQP7 expression was shown to be decreased in response to TNFα [46,48]. Other AQPs permeable to glycerol including AQP3, AQP9 [24], AQP10 [25], and AQP11 [14,42] in human adipocytes and AQP3, AQP9 and AQP11 in mouse adipocytes could participate to the overall glycerol flux. Translocation of both AQP3 and AQP7 from the cytoplasm to the plasma membrane in response to the lipolytic stimulation of beta-adrenergic receptors in murine [49] and human [24] adipocytes highlight the possible relevance of these aquaglyceroporin in glycerol efflux following lipolysis. The differential expression of AQPs in human morbid obesity and obesity-associated T2D may reflect compensatory mechanisms facilitating the glycerol release from adipose tissue and a reduced glycerol influx into hepatocytes to prevent the excessive lipid accumulation and further aggravation of hyperglycemia [24]. Insulin decreased AQP7 in 3T3-L1 adipocytes [45] and increased AQP7 and AQP3 expression in human omental adipocytes [24]. Leptin increased AQP3 levels, whereas it decreased AQP7 levels in human omental adipocytes [24]. Based on its localization and regulation by lipogenic (insulin) and lipolytic (leptin) signals, AQP7 is considered to participate in the control of lipid accumulation, while AQP3 is considered to promote glycerol efflux in response to lipolytic stimuli, such as isoproterenol or leptin [24]. Given the implication of AQPs in glycerol metabolism and whole-body fat mass, AQPs have been elected as potential drug targets in obesity therapy [50] and metabolic syndrome [40]. Identification of the LPS-induced signaling pathway associated with the regulation of AQP3, AQP7 and AQP11 expression in adipocytes may rapidly lead to potential clinical applications. We showed that LPS induced the modulation of AQPs expression by mechanisms involving TLR4 receptor activation. TLR4 can activate the MAPK signaling pathway involving ERK, JNK and p38. ERK is activated in response to growth factors and intracellular calcium increases, for example, while JNK and p38 are activated by a variety of cellular oxidative stresses, such as inflammatory cytokines and heat shock proteins [51,52,53]. Activated JNK phosphorylates a variety of transcription factors which are involved in the formation and activation of the activator protein-1 (AP-1) complex [54]. NFκB activation is involved in inflammation, immunity, cellular proliferation, and apoptosis [55,56]. NFκB activation can be triggered by several factors, including pathogen exposure, inflammatory cytokines, radiation, and other stress signals [57,58]. Activation of TLR4 also triggers signaling pathways leading to both IκBα phosphorylation (IκBα-p) and degradation, as well as NFκB phosphorylation (NFκB-p) and activation [59].

We showed for the first time that JNK and NFκB signaling pathways are involved in the LPS-induced increase in AQP3 mRNA levels and in the LPS-induced decrease in AQP7 mRNA levels in DC, while only NFκB signaling pathway is involved in the LPS-induced decrease in AQP11 (Figure 4). Therefore, we speculate that LPS-induced decrease in AQP7 and AQP11 expression resulting from TLR-4 activation involving JNK and/or NFκB pathways would likely promote lipid accumulation, while LPS-induced increase in AQP3 expression via similar signaling pathway would promote glycerol efflux (Figure 4).

Similarly to AQP7, AQP11 expression is up-regulated following adipocyte differentiation but decreased in LPS-treated DC. In contrast, there was no change in AQP3 expression following adipocyte differentiation while LPS induced an increase of its expression. In addition, distinct subcellular localization adipose aquaglyceroporins [14,24] suggests a probable difference in the role played by each aquaglyceroporin in glycerol metabolism and thereby in obesity. Further studies are required to elucidate the role played by each adipose aquaglyceroporin in both glycerol metabolism and obesity.

In conclusion, several AQPs (classical AQPs as well as aquaglyceroporins) are expressed in murine 3T3-L1 adipocytes differentiated into adipocytes (DC). Differential expression of some AQPs (AQP4, AQP7 and AQP8), specifically expressed only in DC, suggests their role in adipocyte glycerol metabolism. LPS exposure of DC led to decreased AQP7 and AQP11 mRNA levels but increased AQP3 mRNA levels, resulting from TLR-4-induced activation of JNK and/or NFκB pathways. These modifications in aquaglyceroporins expression may be suggestive of an adaptive/protective response to cellular stress involving glycerol metabolism modification, leading to global glycerol/TAG homeostasis alteration.

## 4. Materials and Methods

### 4.1. Reagents

Dulbecco’s modified Eagle’s medium (DMEM, 4.5 g/L glucose), streptomycin/penicillin, fetal bovine serum, horse serum and calf serum were obtained from Invitrogen (Carlsbad, CA, USA). Bovine insulin, 3-isobutyl-1-methylxanthine (IBMX), dexamethasone and LPS were purchased from Sigma (St. Louis, MO, USA). Inhibitors of Toll-like receptor 4 (TLR4) signaling inhibitor (CLI-095), inhibitors of nuclear factor kappa B α (IκBα) (BAY11-7082), c-Jun N-terminal kinases (JNK) (SP600125) and p38 Mitogen-activated protein kinases (MAPK) (SB203580) were purchased from Invivogen (San Diego, CA, USA).

### 4.2. Cell Culture

3T3-L1 murine pre-adipocyte cells were provided by I. Pirson [60] and grown in DMEM supplemented with 10% calf serum, 200 U/mL penicillin and 200 U/mL streptomycin, and in 8% CO_2_ humidified atmosphere at 37 °C, until confluence (UDC). Adipocyte differentiation was induced 2 days post-confluence by incubating cells for 60 h in DMEM supplemented with 10% fetal bovine serum and containing 500 µmol/L IBMX, 0.25 µmol/L dexamethasone and 10 µg/mL insulin, as previously described [41]. The cells were then maintained in the culture medium supplemented with insulin only and this media was changed every 2 days (day 5 and 7) until complete differentiation (monitored by lipid droplet accumulation under the microscope and confirmed by Oil Red Staining) had occurred (day 9). On day 9, DC were treated for 4 h with water (CTL) or 4 h with 1 µg/mL LPS (LPS). DC were also pretreated for 1 h with 20 µmol/L SB203580, 50 µmol/L SP600125, 10 µmol/L BAY11-7082, or 3 µmol/L CLI-095 prior to 4 h CTL or LPS treatment in the presence of inhibitor.

### 4.3. RNA Isolation

Cells were harvested by scrapping in 1 mL acid phenol/guanidinium thiocyanate solution (Purezol, Bio-Rad Laboratories, Hercules, CA, USA), and stored at −20 °C until RNA extraction. RNA extraction was carried out using the Aurum Total RNA Fatty and Fibrous Tissue kit (Bio-Rad Laboratories, Hercules, CA, USA) according to the manufacturer’s instructions, with some minor modifications necessary due to the high lipid content [61]. RNA concentration and purity, as well as RNA integrity, were assessed as previously described [61].

### 4.4. Primer Design

cDNA Synthesis and qPCR Primers design (Table 2), cDNA synthesis and qPCR were performed as previously described [61].

### 4.5. Gene Expression Determined by RT-qPCR

RT-qPCR were performed as previously described [62]. Gene expression stability analysis and matching statistics were performed using Biogazelle qBASE Plus software [63]. Data were normalized using 3 references genes: tyrosine 3-monooxygenase/tryptophan 5-monooxygenase activation protein, ζ polypeptide (mmYWHAZ), Non-POU-domain containing octamer binding protein (mmNONO) and β-actin (mmACTB) that were previously validated for this cellular and experimental system [61].

### 4.6. Statistical Analysis

Data were presented as mean ± S.E.M. of 3 experiments. Group means were compared by repeated measure ANOVA and *t-*test for unique sample. Differences were considered statistically significant at *p* < 0.05. All statistical analysis were performed using SPSS 22 (version 22.0.0.0).

## Figures and Tables

**Table 1:** Real-time quantitative polymerase chain reaction (RT-qPCR) *C*t values of aquaporins (AQPs) expressed in undifferentiated 3T3-L1 cells (UDC; preadipocytes) and 3T3-L1 cells differentiated into adipocytes (DC; adipocytes).

Gene Symbol	*C*_t_ Values		*p* Value
	UDC	DC	
AQP1	29.24 ± 0.81	24.86 ± 0.03	0.01
AQP2	35.96 ± 1.86	35.40 ± 0.96	0.67
AQP3	33.54 ± 1.11	31.34 ± 0.06	0.08
AQP4	ND	31.04 ± 0.97	-
AQP5	ND	ND	-
AQP6	ND	ND	-
AQP7	ND	24.66 ± 0.09	-
AQP8	ND	27.66 ± 0.45	-
AQP9	36.25 ± 0.72	36.24 ± 0.67	0.99
AQP11	30.92 ± 0.13	28.82 ± 0.12	0.0001
AQP12	ND	ND	-

UDC and DC were obtained as described under Material and Methods. Cycle thresholds (*C*_t_) were determined in duplicate for each sample. Data are expressed as *C*_t_ values and are the mean ± S.E.M. (*n* = 3). ND: not detected.

**Table 2:** Real-time quantitative polymerase chain reaction (RT-qPCR) primer sequences.

Genes	Primer Sequence (5′ ≥ 3′)	GenBank Accession Number
*mmAQP1*	Forward: CCGAGACTTAGGTGGCTCAG Reverse: CCCACCCAGAAAATCCAGTG	NM_007472.2
*mmAQP2*	Forward: CATTGGTTTCTCTGTTACCCTG Reverse : AGAAGACCCAGTGATCATCAAAC	NM_009699.3
*mmAQP3*	Forward: GGGCTTCAATTCTGGCTATG Reverse: GAAGACACCAGCGATGGAAC	NM_016689.2
*mmAQP4*	Forward: CCAGCTCGATCTTTTGGACCCGC Reverse: GCTGCGCGGCTTTGCTGAAG	NM_009700.2
*mmAQP5*	Forward: TCTTGTGGGGATCTACTTCAC Reverse: AGAAGTAGAGGATTGCAGCC	NM_009701.4
*mmAQP6*	Forward: GGCTATGGCCTATATCGCTG Reverse: GCCAGTTGATGTGCTGTTG	NM_175087.4
*mmAQP7*	Forward: GCTGCTTCAGGTCCACCCACAAC Reverse: GCCACGGAACCAAGGCCAAACAC	NM_007473.4
*mmAQP8*	Forward: ATGGCTGGCTACTGGGACTT Reverse: CGCCAGCAGTTCTTCTTCAC	NM_007474.2
*mmAQP9*	Forward: TATCCCCAGAAGCCCAAACT Reverse: GCTGTTGGGATCAAACTGGA	NM_022026.3
*mmAQP11*	Forward: TTGCTCCTTCTGTAGGTGTG Reverse: ACTGTCCTGGGACTTAGTTC	NM_175105.3
*mmAQP12*	Forward: GCCCTACACATCTGCCTTC Reverse: GAGGACAGCCAGGATCATC	NM_177587.2
*mmNONO*	Forward: TGCTCCTGTGCCACCTGGTACTC Reverse : CCGGAGCTGGACGGTTGAATGC	NM_023144.2
*mmACTB*	Forward: CCTGTGCTGCTCACCGAGGC Reverse : GACCCCGTCTCCGGAGTCCATC	NM_007393.3
*mmYWHAZ*	Forward: AAAAACAGCTTTCGATGAAGCC Reverse : GCCGGTTAATTTTCCCCTCC	NM_011740.3

## Abbreviations

AQP	Aquaporin
CT	Control
DC	3T3-L1 cells differentiated into adipocytes
IκBα	Inhibitors of nuclear factor κ B α
JNK	c-Jun N-terminal kinases
LPS	Lipopolysaccharide
NFκB	Nuclear factor-κB
TLR4	Toll-like receptor 4
UDC	Undifferentiated 3T3-L1 cells
